# Electrical Bioimpedance-Based Monitoring of Intracochlear Tissue Changes After Cochlear Implantation

**DOI:** 10.3390/s24237570

**Published:** 2024-11-27

**Authors:** Leanne Sijgers, Marlies Geys, Gunnar Geissler, Patrick Boyle, Alexander Huber, Flurin Pfiffner

**Affiliations:** 1 Department of Otorhinolaryngology, Head & Neck Surgery, University Hospital Zurich, University of Zurich, 8091 Zurich, Switzerland; 2 European Research Center, Advanced Bionics GmbH, 30625 Hannover, Germany

**Keywords:** digital biomarkers, remote hearing healthcare, cochlear implant, neuroprosthetics, hearing technology, auditory rehabilitation

## Abstract

Background: This study examined electrical bioimpedance as a biomarker for intracochlear tissue changes after cochlear implant surgery, comparing monopolar, three-point, and four-point impedance measurements over time and evaluating different measurement systems and approaches. Methods: Impedance measurements were obtained from 21 participants during surgery and at four postoperative stages. Monopolar impedances were recorded using the Bionic Ear Data Collection System (BEDCS) and the Active Insertion Monitoring (AIM) system. Three- and four-point impedances were recorded directly using BEDCS, and indirect three-point impedances were additionally derived from Electrical Field Imaging matrices recorded using BEDCS or AIM. Results: There was an 11% relative error between monopolar measurements from BEDCS and AIM and a 25% discrepancy between direct and indirect three-point measurements. Despite this, direct and indirect measurements from both systems were useful for tracking postoperative impedance shifts. Three- and four-point measurements showed a strong relationship both during and after surgery. Our results suggest that three- and four-point measurements are more specific than monopolar impedances in capturing localized tissue changes. Conclusions: Three- and four-point impedance measurements are potential markers of intracochlear tissue changes over time. While direct three-point impedance measurements offer higher accuracy, indirect measurements provide a feasible alternative for monitoring intracochlear changes in clinical settings lacking the option of direct measurements.

## 1. Introduction

Cochlear implants (CIs) are neuroprosthetic devices that can restore hearing by electrically stimulating the auditory nerve using an electrode array that is surgically inserted into the inner ear (cochlea). Typically, they consist of 12 to 22 intracochlear electrode contacts and one or two extracochlear electrodes, which can be used as far-field references for monopolar stimulation at each intracochlear contact. CIs have been successfully used for the last forty years as a solution for people with severe to profound sensorineural hearing loss for whom sound amplification by hearing aids does not provide sufficient benefit [1].

However, hearing outcomes with CIs vary greatly, with some recipients attaining excellent speech understanding even in noisy acoustic environments, while others achieve only minimal understanding in quiet settings. The factors affecting an individual’s hearing performance are numerous and a topic of ongoing investigation [2]. For example, speech recognition outcomes in adults are positively influenced by greater preoperative acoustic hearing and previous hearing aid use [3,4], while factors such as partial electrode array insertion [5], prolonged duration of severe hearing loss [4,6], and older age [7,8] tend to negatively impact performance.

Nowadays, many CI recipients have residual acoustic hearing prior to receiving their implants. While this acoustic hearing is no longer sufficient to realize adequate speech understanding with a hearing aid, it can help with speech perception and music appreciation when combined with electric hearing through the CI [9,10]. To this end, acoustic hearing should be preserved during and after the surgical insertion of the CI’s electrode array into the cochlea, and strategies to monitor and preserve hearing during surgery are a topic of current investigation [11,12,13,14,15,16,17,18,19,20,21]. Unfortunately, the formation of a fibrotic tissue sheath around the array and intracochlear neo-ossification commonly occur after CI surgery [22,23]. These reactions are thought to be triggered by trauma caused by the surgical intervention of electrode array insertion into the cochlea [24] and resemble the wound healing responses observed in other tissues [25]. Various studies have demonstrated that intracochlear tissue formation disrupts acoustic hearing [26,27,28] and may also affect electric hearing with the CI, although the impact on electric hearing is thought to be minimal [29]. Moreover, this tissue growth may complicate future surgical procedures, including the removal and replacement of electrodes, and may hinder future therapeutic approaches, such as the regeneration of hair cells and neurons. Furthermore, because stimulation with a cochlear implant is current-controlled, the higher voltage required due to this tissue growth leads to increased power consumption, potentially impacting device performance and longevity.

Bioimpedance measurements recorded at a CI’s intracochlear electrode contacts are potential biomarkers of fibrotic tissue growth [30,31,32,33,34]. Impedances typically rise following cochlear implantation, with greater increases observed at the base than at the apex [35]. With the advancement of digital healthcare systems that enable remote patient monitoring over time [36], bioimpedance measurements are well positioned to detect intracochlear tissue changes following CI implantation. This could facilitate targeted interventions, such as the timely administration of drugs to reduce inflammation and new tissue formation [37,38,39].

Impedance measurements in a CI are typically conducted in monopolar mode. In this setup, stimulation occurs between an intracochlear electrode and an extracochlear reference electrode, with the resulting electrical potentials also being recorded between these same contacts. In this configuration, different types of stimuli are commonly used. Electrochemical impedance spectroscopy, which uses alternating current swept over multiple frequencies, can identify complex impedance at each frequency [40,41]. The frequency response function of impedance can also be characterized with alternative stimuli, such as current pulses, albeit with a lower signal-to-noise ratio. Given that standard CI stimulation uses biphasic electric stimuli, recorded waveforms are often described in terms of ‘impedance components’, specifically access resistance (R_a_) and polarization impedance (Z_p_), rather than characterizing the entire frequency response function. R_a_ represents the resistance at the start of the pulse and primarily reflects the tissue impedance between the recording electrodes, while Z_p_ arises from the electrode contact’s charging during a pulse and includes both capacitive and resistive components [42]. An increase in R_a_ has been associated with inflammation and fibrotic tissue development in animal studies [43,44], and loss of acoustic hearing in human CI recipients [45].

As an extension of monopolar impedance measurements, an Electrical Field Imaging (EFI) matrix can be recorded [46]. In this process, all intracochlear electrode contacts are stimulated sequentially in monopolar mode. In contrast to the conventional approach of only recording the resulting potentials at the stimulating contacts, the voltage response is recorded in monopolar configuration along all intracochlear electrodes on the electrode array.

Impedances can also be recorded in bipolar mode, whereby both stimulation and recording occur between two neighboring intracochlear electrodes, potentially making the measurement more specific in capturing local impedance variations. Recently, two variations of bipolar impedance measurements have been introduced: three-point impedance measurements were proposed by our group [47], and four-point impedance measurements were introduced by Bester et al. [48].

In both of these experimental measurements, the electric potentials are recorded between two neighboring intracochlear electrodes, similar to bipolar recordings. However, the recording electrodes are not used for stimulation, which helps to avoid confounding effects from the voltage component induced at the electrode–tissue interface of the stimulating electrode contact. Three-point impedance measurements are essentially bipolar impedances recorded in response to monopolar stimulation at a neighboring electrode [47], while four-point impedance measurements are obtained by passing current between two outer electrodes and measuring the potential between two inner electrodes [48]. A recent study involving human CI recipients demonstrated that the timescale of four-point impedance changes after implantation matches intracochlear fibrosis formation [33].

Although CI manufacturers have enabled monopolar and EFI recordings in commercial systems, standard clinical systems are not yet equipped to perform three-point and four-point impedance measurements. However, three-point impedances can be deduced from EFI recordings, provided that the recording gains are high enough to ensure a sufficient signal-to-noise ratio. They are derived by subtracting the potential measured two electrodes basal to the stimulating electrode from the potential measured one electrode basal to the stimulating electrode and dividing the resulting potential by the input current [47]. Since many clinics have large EFI datasets available for retrospective analysis, derivation of three-point impedances from these matrices could facilitate new insights on intracochlear tissue changes post-implantation. For this purpose, the validity of indirect three-point impedances, derived from EFI matrices measured using clinical systems, should be evaluated by comparing them with direct three-point impedance recordings.

The aims of the present study were twofold. First, we evaluated recordings performed with different measurement systems and compared indirect three-point impedances derived from EFI matrices with direct three-point impedance recordings. Second, we assessed how impedances change over time after implantation and compared monopolar, three-point, and four-point impedance measurements in CI users.

## 2. Materials and Methods

### 2.1. Materials

Participants received CIs with either a HiFocus^TM^ MidScala or a HiFocus^TM^ SlimJ electrode array from Advanced Bionics LLC, Valencia, CA, USA. Each array features 16 intracochlear electrode contacts, with electrode number 1 being the most apical (most deeply inserted) electrode. The arrays are designed for different intracochlear placements: MidScala arrays target the middle of the cochlear duct, without touching either the lateral (outer) wall or the modiolus (internal wall), whereas SlimJ arrays aim for a position closer to the cochlea’s lateral wall to optimally protect the intracochlear structures.

When conducting electrical impedance measurements via the CI, the electrodes’ recorded potentials can be amplified using the CI’s internal amplifier prior to being digitized by an analog-to-digital converter, which can sample at 56 kHz with a 9-bit resolution. The digitized signal can be transmitted to a recording device through an external coil on the skin, using electromagnetic induction. We used two recording devices from Advanced Bionics LLC, designed for connectivity with their CIs. One is a research platform named Bionic Ear Data Collection System (BEDCS), version 1.18, which was controlled using customized MATLAB (R2015a; MathWorks, Inc., Natick, MA, USA) scripts. The platform offers flexibility in defining stimulation and recording parameters and was used to record direct three-point and four-point impedances as well as EFI matrices. The other device is a clinical system named Active Insertion Monitoring (AIM), with which EFI matrices were collected.

Data processing and parameter extraction from impedance measurements were conducted using MATLAB (R2022b), and statistical analysis was performed with R version 4.3.1.

### 2.2. Measurement Protocol and Parameter Extraction

Adult CI recipients were prospectively enrolled in the study at the University Hospital Zurich. The study was performed with the approval of the Ethical Committee of Zurich (KEK-2022-02279) and in concordance with the Declaration of Helsinki. A total of 21 individuals participated and provided written informed consent.

Impedance measurements were scheduled at five different timepoints:

- During CI surgery, after full insertion of the CI electrode array (intraop);
- Approximately six weeks post-surgery (postop 1);
- Approximately three months post-surgery (postop 2);
- Approximately six months post-surgery (postop 3);
- Approximately one year post-surgery (postop 4).

The following impedance measurements were collected:

- EFI with BEDCS;
- EFI with AIM;
- Three-point impedance with BEDCS;
- Four-point impedance with BEDCS.

Monopolar R_a_ and indirect three-point impedance recordings were deduced from the EFI matrix measured with BEDCS or AIM, as described in [47]. Since the impedance recordings were part of an ongoing study, which involved various different measurements, not all impedance recordings were conducted at all timepoints in every participant. Table A1 in Appendix A provides an overview of the parameters available for each participant. Impedance recordings obtained at individual electrodes were excluded from analysis in the case of open circuits (impedances > 20 kΩ), for example, due to air bubbles on the electrode contacts.

The stimuli used to obtain impedance recordings consisted of cathodic-first biphasic waveforms, as is standard for stimulation with a CI. The current amplitude was set at 32 μA for AIM and 34 μA for BEDCS. For the EFIs recorded with AIM, a measurement gain of 1 was applied to recordings above 11.9 kΩ, whereas a gain of 6 was used for impedances between 4 and 11.9 kΩ and a gain of 18 was applied to recordings below 4 kΩ, as is common for recordings made at the non-stimulating electrodes. Depending on the implant’s tank voltage, the effective gain for these recordings is typically slightly lower, ranging between 16 and 18.

BEDCS EFI recordings were made with a measurement gain of either 1 or 3 for the stimulating electrode and a constant gain of 18 for the non-stimulating electrodes. The direct three- and four-point impedance recordings were made with a measurement gain of either 100 or 300, depending on the recorded voltage. Responses to 10 stimuli were averaged for each recording made with BEDCS, while no averages were made for recordings made with AIM.

Following biphasic stimulation, the impedance values were calculated by first taking the initial sample of the recorded negative voltage waveform and subtracting the preceding sample’s value from it. This result was then divided by the input current.

In some cases, multiple recordings were made using different recording modalities to enable a comparison of impedance measurements obtained using different measurement systems and methods. To assess impedance variations after surgery and to compare the various types of impedance recordings, such as monopolar and three-point impedances, recordings using BEDCS were preferred over those obtained with AIM. Furthermore, direct three-point impedance measurements were used instead of indirect ones in these assessments.

### 2.3. Measurement System and Method Comparison

A comparison between monopolar R_a_ recordings performed with BEDCS and AIM, as well as between the direct and indirect three-point impedance measurements, was conducted through a Bland-Altman analysis [49]. This analysis involved plotting the difference between the two measurements against their average and calculating the limits of agreement, defined as the mean difference ± 1.96 standard deviations of the difference.

To quantify the bias in the R_a_ and three-point impedance recordings, the mean difference between the measurements obtained from the two techniques in each comparison was calculated. The relative error was determined as the absolute value of the bias divided by the average measurement obtained from the standard recording (considered as BEDCS for R_a_ measurements and direct recording for three-point impedance measurements), expressed as a percentage.

### 2.4. Impedance Measurement Analysis

Linear mixed regression (LMR) was performed using R’s lme4 toolbox [50] to assess the relationship between monopolar and three-point impedance and between three- and four-point impedance. Regression parameters were obtained using the maximum likelihood estimator. Electrodes 1 and 16 were excluded from analysis of the measurement results, as three- and four-point impedances were not measured for these contacts. For the analysis of the three- vs. four-point impedance measurements, electrode 15 was additionally excluded because four-point impedances were not measured for this contact.

As fixed effects, impedance recordings and electrode number were included as numeric variables, while the measurement number was included as a factor variable. Impedance recordings were scaled prior to inclusion to improve model convergence. Additionally, the three- and four-point impedance recordings were log-transformed, using the natural logarithm, to reduce skewness in the data and straighten the models’ Q-Q residual plots.

Initially, all fixed effects were also considered as potential random effects, grouped by participant, to discern the most suitable model based on Akaike Information Criterion (AIC) values. Random effects were excluded if either their removal yielded a model with a lower AIC value or it helped in achieving model convergence. This process confirmed the necessity of a random effect of electrode on the relation between monopolar and three-point impedance and a random effect of three-point impedance on the relation between three- and four-point impedance. An unstructured covariance matrix was used to model the random effects. The equations of the resulting LMR models, expressed in Wilkinson notation, are presented in Equations (1) and (2).

log(3PI_scaled) ~ Ra_scaled + measurement + electrode + (1 + electrode | participant)(1)

log(4PI_scaled) ~ log(3PI_scaled) + measurement + electrode + (1 + log(3PI_scaled) | participant)(2)

The significance of the fixed effects was assessed by *t*-tests using Satterthwaite’s method (lmerTest toolbox [51]). The marginal and conditional R^2^ were calculated using the MuMIn toolbox [52].

For the analysis using the model in Equation (2), data from the third and fourth postoperative sessions were not available. The models in Equations (1) and (2) were additionally fitted to only the postoperative impedance data to assess the influence of tissue alterations on the relationships between impedance measurements.

## 3. Results

### 3.1. Data Overview

Table 1 presents the demographics, etiological factors, and surgical details of the study participants. For participant 1, a cochleostomy approach (drilling an opening anterior and inferior to the round window) was used to insert the electrode array due to scar tissue found in the round window niche. For the rest of the participants, insertion was performed through the cochlea’s round window.

During CI surgery, a full electrode array insertion was achieved for all participants. Postoperative imaging revealed a tip fold-over at electrode 4 in participant 3, meaning that the array folded onto itself during insertion rather than achieving a straight placement within the cochlea. In participants 12 and 17, the electrode array migrated after insertion, resulting in five and three extracochlear electrodes, respectively. In addition, electrode contact 4 in participant 12 showed an open circuit, and therefore, three-point impedances could not be measured for electrodes 3, 4, and 5. Postoperative data from the extracochlear contacts in participant 12 were also omitted from the analysis due to open-circuit issues. For participants 3 and 17, impedances were measurable for all electrode contacts and were included in the analysis.

### 3.2. Measurement System and Method Comparison

**Figure 1:**
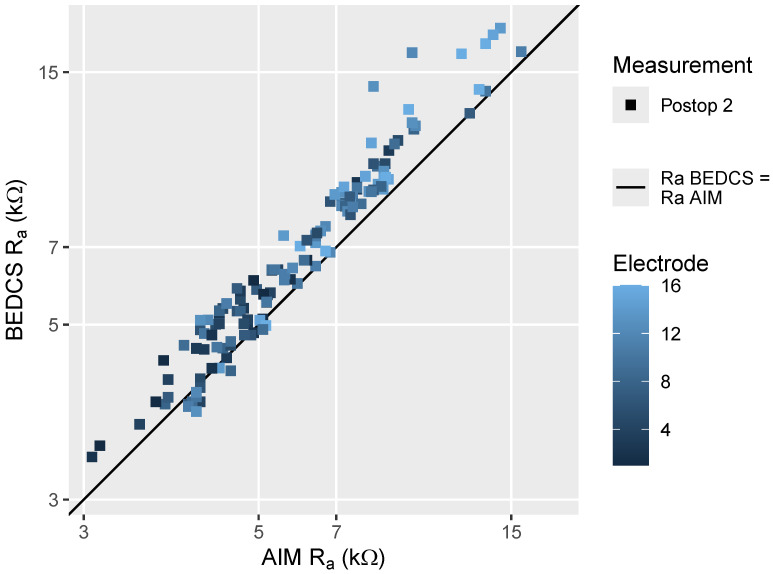
Comparison of the monopolar access resistance (R_a_) measurement conducted using BEDCS and AIM, using color coding to indicate the electrode number. All recordings were obtained during the second postoperative recording session.

**Figure 2:**
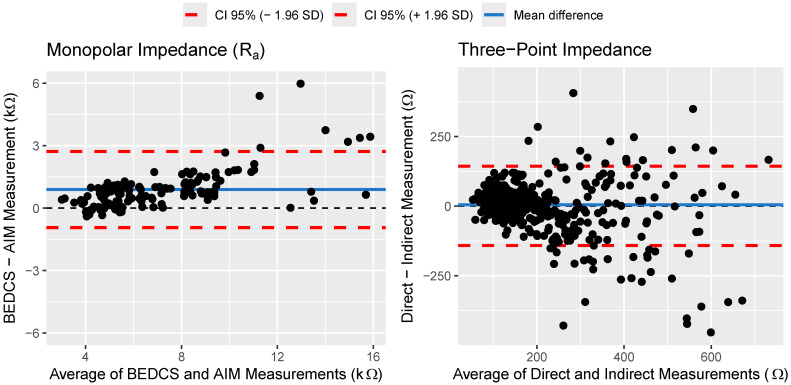
Bland–Altman plots for the monopolar impedance measurement conducted using BEDCS and AIM (**left**) and the direct and indirect three-point impedance measurements (**right**).

Figure 1 illustrates the comparison between monopolar R_a_ measurements obtained using BEDCS and AIM. They cover the range between 3 and 18 kΩ with basal (higher-numbered) electrodes showing higher impedances overall. The BEDCS measurements are generally slightly higher than those recorded via AIM, particularly in the range of 9 to 12 kΩ. This is confirmed by the Bland–Altman analysis shown in the left panel of Figure 2, which revealed a mean bias of 0.89 kΩ, with limits of agreement of ±1.83 kΩ around the mean difference. Overall, the data from the two recording systems demonstrate a high degree of concordance, with a relative error of 11%.

**Figure 3:**
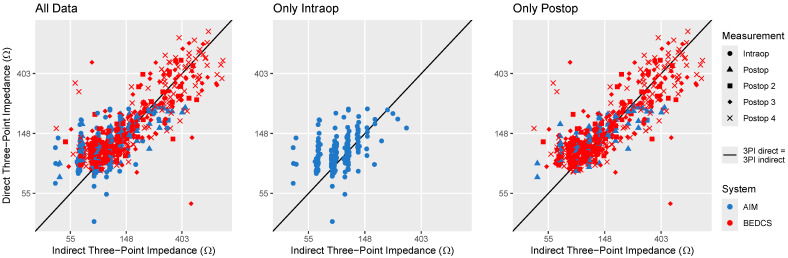
Comparison of the direct three-point impedance measurement conducted using BEDCS, and the indirect measurement derived from the EFI matrix recorded using BEDCS or AIM. Symbols are used to indicate the recording session, while the measurement system is color-coded. In cases where EFI recordings were conducted using both BEDCS and AIM, the indirect three-point impedances were deduced from the BEDCS recordings.

The comparison between the direct and indirect three-point impedance recordings, shown in Figure 3, revealed a larger spread. In particular, the indirect intraoperative recordings, represented in the middle panel of Figure 3, exhibit noticeable quantization steps and are affected by noise. This makes the indirect measurements unsuitable for comparing low three-point impedance values with each other, as is usually the case when solely looking at intraoperative data. However, a clear relationship between direct and indirect recordings emerges when including postoperative data, where impedances are known to increase strongly due to fibrotic tissue formation in some cases. Here, intraoperative three-point impedances ranged from 35 to 310 Ω, while postoperative values increased up to around 840 Ω. Despite variability in the indirect recordings, they reliably differentiate between higher and lower three-point impedances, making them useful for detecting large impedance shifts over time. The Bland–Altman analysis shown in the right panel of Figure 2 revealed only a very minimal bias of 4.6 Ω, with limits of agreement of ±143 Ω around the mean difference. The relative error in the indirect three-point impedance recordings was 25% on average.

### 3.3. Analysis of Impedance Measurements and Variability

**Figure 4:**
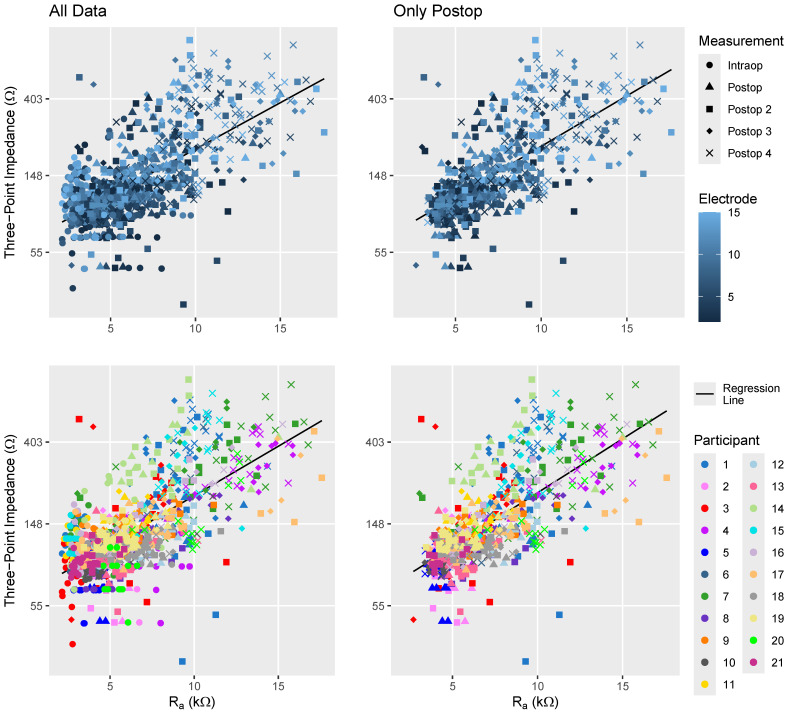
Comparison of monopolar R_a_ and three-point impedance measurements. Symbols are used to indicate the recording session, with the upper plots employing color coding to indicate electrode number and the lower plots using color coding to differentiate participants. Note that the three-point impedances are displayed on a logarithmic scale, while the *x*-axis depicting R_a_ is linear, in alignment with model 1. In cases where recordings were obtained using multiple systems or methods, recordings with BEDCS were used instead of recordings made with AIM, and direct three-point impedance measurements were favored over indirect recordings.

A comparison between monopolar and three-point impedance recordings is illustrated in Figure 4. There is a consistent trend towards higher three-point impedance values being associated with larger monopolar impedances. The LMR analysis using model 1, shown in Table 2, confirms this finding by demonstrating a positive relationship between these two types of impedance measurements. For this analysis, the three-point and monopolar impedance (R_a_) values were scaled by dividing by the standard deviation obtained from the dataset used for this analysis—110 Ω for three-point impedance and 2.93 kΩ for monopolar impedance values.

The upper section of Figure 4 indicates that the higher electrode numbers (positioned towards the base) and later measurements tended to have higher monopolar and three-point impedances than more apical electrodes. The LMR analysis using model 1 indicated that measurement timing and electrode number influenced the impedance values differently; specifically, later measurements and more basal electrodes exhibited a disproportionately higher increase in three-point than monopolar impedances, as indicated by the model parameters in Table 2.

The lower section of Figure 4 indicates variability among participants, with individuals such as participants 7, 14, and 15 showing generally higher three-point impedance relative to the general trend between three-point and monopolar recordings, while participants 17 and 20 exhibited lower three-point impedance. The incorporation of participant-dependent effects into model 1 greatly increased the model’s R^2^ values, as shown in Table 3. This enhancement in R^2^ was even stronger when including only postoperative data and indicates that the relationship between the monopolar and three-point impedances depends on participant-specific factors.

**Figure 5:**
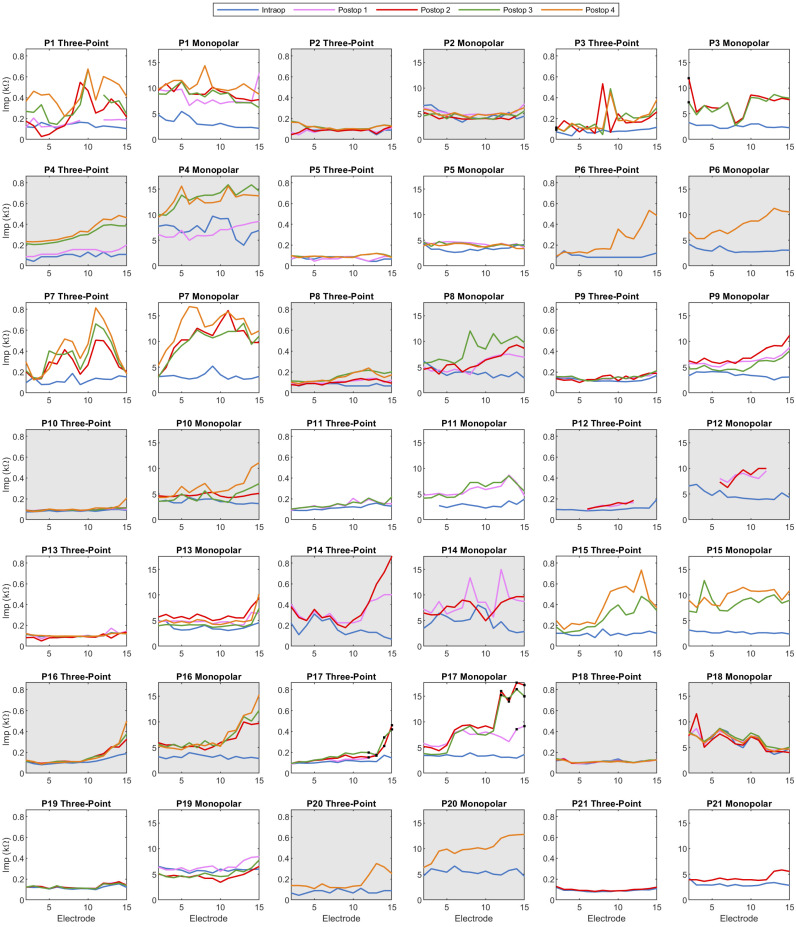
Visualization of the monopolar and three-point impedance measurements against electrode number for each participant and recording session, indicated using color coding. In cases where recordings were obtained using multiple systems or methods, recordings with BEDCS were used instead of recordings made with AIM, and direct three-point impedance measurements were favored over indirect recordings. Participant 3 had a tip fold-over at electrode 4, while participants 12 and 17 had five and three extracochlear electrodes, respectively. Consequently, specific electrodes were deactivated in these participants’ implant settings, indicated by black dots. For participant 12, the deactivated electrodes had open circuits and are thus not depicted in the figure. Additionally, a few recordings from participant 1 were affected by amplifier saturation and were therefore excluded from the figure.

Figure 5 shows the monopolar and three-point impedance recordings for each participant at all recording timepoints. In cases such as those of participants 2 and 5, where impedance levels are consistently low with minimal variation across the array, the impedance patterns from both types of recordings align closely. Both recording methods also detect typical increases in basal electrode impedance, as in participants 6, 8, and 10. However, three-point impedance recordings often show more regional variations than monopolar recordings. Specifically, in the cases of participants 15, 16, 17, and 20, three-point impedance increases were predominantly observed at the base, in contrast to the broader increases recorded by monopolar measurements along the entire electrode array. In addition, in participant 7, a dip in the middle of the electrode array was reflected in three-point but not monopolar impedance. Moreover, for participants 3 and 17, monopolar impedances increased for all deactivated electrodes (in Figure 5, electrode 2 in participant 3 and all deactivated electrodes in participant 17), whereas three-point impedances exhibited large increases only for clusters of deactivated neighboring electrodes (in Figure 5, only for electrodes 14 and 15 in participant 17). The tip fold-over in participant 3 was most visible in the three-point impedance, albeit more basal than the location of the fold-over itself, which occurred at electrode 4.

**Figure 6:**
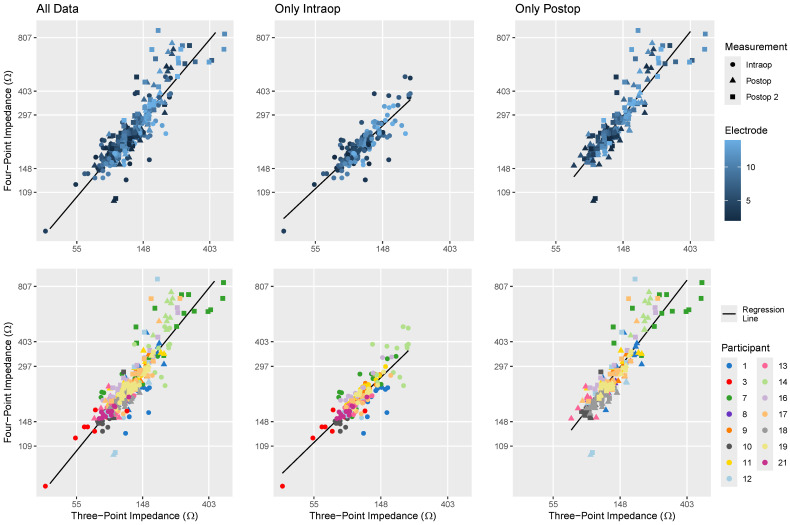
Comparison of three- and four-point impedance measurements, including only direct recordings made with BEDCS. Symbols are used to indicate the recording session, with the upper plots employing color coding to indicate electrode number and the lower plots using color coding to differentiate participants. Both the *x*- and *y*-axes use a logarithmic scale, in alignment with model 2.

Figure 6 compares the direct three- and four-point impedances obtained using BEDCS, showing a strong association between the two measurements. This association is also supported by the LMR analysis using model 2 presented in Table 1 and the high R^2^ values listed in Table 2. In this analysis, both impedance measurements were scaled by dividing by their respective standard deviations obtained from the dataset used for this particular analysis—53 Ω for three-point impedance and 121 Ω for four-point impedance values. Figure 6 does not clearly show a visual relationship between electrode number and impedance. However, it reveals that the agreement between three- and four-point impedance measurements weakened for participants with higher impedance values, such as participants 7 and 14. The LMR analysis using model 2 indicated that later measurements exhibited disproportionately higher increases in four-point than three-point impedances, as indicated by the model parameters in Table 2. The effect of electrode was minimal and became significant only when intraoperative data were omitted. Moreover, according to R^2^ values in model 2 (Table 3), participant-specific factors had a greater impact on the relationship between three- and four-point impedances postoperatively than when also including intraoperative data.

## 4. Discussion

### 4.1. Measurement System and Method Comparison

In this study, we recorded monopolar R_a_ using two distinct systems, BEDCS and AIM, and observed a relative error of 11% between them. Additionally, the analysis revealed a bias of 0.89 kΩ and limits of agreement of 1.83 kΩ surrounding the mean difference. Considering that the measurement range spanned from 3 to 18 kΩ, these discrepancies are minor. Therefore, within the context of our study, we deem the differences between R_a_ recordings with the two systems clinically insignificant, supporting our rationale for the interchangeable application of both systems without compromising the integrity of our findings. However, for tracking impedances over time in CI users, it is recommended to consistently use one system to minimize fluctuations in the measurements unrelated to physical changes.

We also employed two methodologies to measure three-point impedances: direct measurements and indirect estimations derived from the EFI matrix. The indirect approach exhibited quantization steps of approximately 20 Ω. In addition, noise from various sources contributed to the error, including the inherent noise floor of the amplifier—the baseline background noise generated by the amplifier’s circuitry in the absence of a signal—and other internal electronic components, as well as external noise sources. Together, these factors resulted in a relative error of 25% between the two methods. While only a minimal bias was present, the limits of agreement of 143 Ω around the mean difference were particularly consequential for distinguishing differences between intraoperative recordings, which exhibited impedance values ranging from 35 to 310 Ω. However, despite the clinically significant discrepancies observed between the two methods, indirect recordings were still useful for identifying electrodes exhibiting substantial impedance increases over time. As postoperative impedance values increased up to around 840 Ω in this study, indirect three-point impedances provided a useful alternative in cases where more precise direct recordings were unavailable. Averaging the results of multiple repeated measurements could help reduce noise in future clinical systems, albeit at the cost of increased measurement time.

### 4.2. Comparison of Monopolar, Three-Point, and Four-Point Impedance

When assessing both the three-point versus monopolar impedances (Figure 4 and model 1) and the comparison between three- and four-point impedances (Figure 6 and model 2), participant-dependent effects became more pronounced in the overall relationship when intraoperative recordings were omitted (Table 3). This may be because fibrotic tissue formation alters the current pathways in a manner that varies across individuals [53], resulting in varying impacts on the different impedance measurements.

The LMR analyses also showed that the recording session significantly influenced the relationships among different impedance measurements. Specifically, for later sessions, the increase in three-point impedance was greater than that in monopolar impedance (model 1) yet smaller than the increase observed in four-point impedance (model 2) when compared to the general relationships and variability in the measurements. This pattern might be related to the precision of the measurements. More precise measurements are likely better at detecting tissue changes over time and are less affected by external variabilities such as noise.

### 4.3. Monopolar and Three-Point Impedance Changes over Time

Research indicates that immediately following insertion of the CI electrode array, electrode placement is the primary factor impacting clinical electrode impedance [54]. Notably, since the round window of the cochlea may act as a current sink at its base [55,56], monopolar impedances tend to increase from the base towards the apex [54], which can be attributed to the extended return path to the ground. However, in the months after CI surgery, this pattern shifts, resulting in higher basal impedances compared to apical ones [33]. This change is likely due to the more pronounced fibrotic tissue development near the cochlea’s entry point for the electrode array [30]. A recent study on four-point impedances in the early postoperative phase showed that they also tend to increase more towards the base than at the apex [34].

Our results suggest that three-point impedance is more specific to localized tissue changes than monopolar impedance measurements. While monopolar impedances generally increased along the entire electrode array, three-point impedances exhibited a mid-array dip or increased values primarily at the basal end in certain instances. This difference may stem from the impact of fibrotic tissue formation at the cochlea’s base, which could also influence more apical monopolar recordings in cases where the stimulation and recording pathways partially pass through the base of the cochlea. Given that three-point impedances are recorded using a bipolar approach, they are less susceptible to alterations occurring further along the electrode array than monopolar impedances [47]. Although not directly evaluated in this study, four-point impedances might offer even greater specificity than three-point impedances due to their more localized manner of stimulation.

### 4.4. Impedance as a Biomarker of Fibrosis

In this study, we have shown that three- and four-point impedance measurements are potential markers of intracochlear tissue changes over time. Previous research indicated that impedance changes measured at the CI’s intracochlear electrodes after surgery can be caused by a multitude of factors, including electrochemical reactions at the electrode–tissue interface [57,58,59], biological deposits (such as protein) on the electrode surface [59], intracochlear bleeding [48], and new tissue formation [31]. New tissue formation in the cochlea can range from a thin fibrous sheath around the electrode array to the development of new bone [60,61,62,63].

The degree of fibrotic tissue formation has been correlated with monopolar R_a_ and complex impedance [31,43], while monopolar Z_p_ has been associated with biological deposits on the intracochlear electrodes [32]. In this study, we showed that three-point impedance recordings often show more regional variations than monopolar recordings, potentially making them more specific to local tissue alterations. We also revealed a strong relationship between three- and four-point impedance measurements. Future laboratory studies are needed to directly investigate the link between three-and four-point impedances and the degree of new tissue development and thereby confirm their validity as biomarkers.

## 5. Conclusions

This study highlights the potential of three- and four-point impedance measurements as markers for monitoring intracochlear tissue changes over time. The higher specificity of these measurements compared to standard monopolar impedance recordings makes them particularly promising for gaining specific insights into tissue alterations along the CI electrode array. Importantly, three-point impedance measurements can either be recorded directly or derived from clinical EFI recordings, provided that the signal-to-noise ratio is sufficiently high. While direct three-point measurements offer superior accuracy, the ability to indirectly deduce these measurements ensures broader applicability in clinical settings where direct measurement tools may be unavailable. Furthermore, the observed high agreement between three- and four-point impedances suggests that they likely capture the same underlying physiological changes, despite some divergence in higher impedance ranges, likely due to the four-point impedance’s more localized stimulation pathway. Together, these findings support the potential of both measurement approaches for advancing future intracochlear monitoring.

## Figures and Tables

**Table 1:** Demographics, etiological factors, and surgical details of the study participants.

Variable	Average or Total of n = 21 Participants
Mean age (years)	63 (range, 28–80)
Gender (%)	
Male	14 (67)
Female	7 (33)
Etiology (%)	
Sudden SNHL	2 (10)
Trauma	1 (5)
Hereditary	1 (5)
Idiopathic	17 (81)
Implantation side (%)	
Left	5 (24)
Right	16 (76)
Electrode array (%)	
MidScala	8 (38)
SlimJ	13 (62)

**Table 2:** Regression parameter estimates of the fixed effects in models 1 and 2, corresponding standard errors (SEs), and *p* values of the *t* tests obtained via Satterthwaite’s degrees of freedom method. Note that for the analyses using model 2, data for the third and fourth postoperative recording sessions were unavailable, leading to their exclusion from the model parameters. The asterisk (*) indicates significance.

Included Data	Parameter	Parameter Estimate	SE	Pr(t)
*(1) log(3PI_scaled) ~ Ra_scaled + measurement + electrode + (1 + electrode | participant)*				
Model 1, all data	Intercept Ra_scaled Measurement: postop 1 Measurement: postop 2 Measurement: postop 3 Measurement: postop 4 Electrode	−0.612 0.221 0.057 0.086 0.231 0.297 0.025	0.061 0.013 0.029 0.028 0.028 0.033 0.004	<0.001 * <0.001 * 0.048 * 0.003 * <0.001 * <0.001 * <0.001 *
Model 1, only postop	Intercept Ra_scaled Measurement: postop 2 Measurement: postop 3 Measurement: postop 4 Electrode	−0.521 0.182 0.042 0.185 0.245 0.033	0.068 0.017 0.029 0.028 0.033 0.006	<0.001 * <0.001 * 0.149 <0.001 * <0.001 * <0.001 *
*(2) log(4PI_scaled) ~ log(3PI_scaled) + measurement + electrode + (1 + log(3PI_scaled) | participant)*				
Model 2, all data	Intercept log(3PI_scaled) Measurement: postop 1 Measurement: postop 2 Electrode	−0.164 0.859 0.104 0.159 0.003	0.048 0.057 0.020 0.024 0.002	0.003 * <0.001 * <0.001 * <0.001 * 0.168
Model 2, only postop	Intercept log(3PI_scaled) Measurement: postop 2 Electrode	−0.172 0.938 0.079 0.009	0.112 0.109 0.025 0.003	0.149 <0.001 * 0.002 * 0.006 *

**Table 3:** Marginal (R^2^m) and conditional (R^2^c) R^2^ of the linear mixed regression models.

Model	R^2^m	R^2^c
1, all data	0.42	0.70
1, only postop data	0.32	0.72
2, all data	0.76	0.83
2, only postop data	0.70	0.88

## Data Availability

The data that support the findings of this study are available from the corresponding author upon reasonable request.

## Appendix A

**Table A1:** Overview of the measurements performed for each participant at the various recording sessions. I stands for intraoperative, while P1 to P4 stand for postop 1 to postop 4. “✓” indicates that a measurement was performed, whereas “-” indicates that it was not.

Participant	AIM Ra					BEDCS Ra					Indirect 3PI					Direct 3PI					Direct 4PI				
	I	P1	P2	P3	P4	I	P1	P2	P3	P4	I	P1	P2	P3	P4	I	P1	P2	P3	P4	I	P1	P2	P3	P4
01	✓	✓	✓	-	-	-	-	✓	-	✓	✓	✓	✓	-	✓	✓	✓	-	-	✓	✓	✓	-	-	-
02	✓	✓	✓	-	-	-	-	✓	-	✓	✓	✓	✓	-	✓	-	-	-	-	✓	-	-	-	-	-
03	✓	-	-	-	-	-	-	✓	-	-	✓	-	✓	-	-	✓	-	-	-	✓	✓	-	-	-	-
04	✓	✓	-	-	-	-	-	-	✓	✓	✓	✓	-	✓	✓	-	-	-	✓	✓	-	-	-	-	-
05	✓	✓	-	-	-	-	-	-	✓	✓	✓	✓	-	✓	✓	-	-	-	✓	✓	-	-	-	-	-
06	✓	-	-	-	-	-	-	-	-	✓	-	-	-	-	✓	-	-	-	-	✓	-	-	-	-	-
07	✓	-	✓	-	-	-	-	✓	✓	✓	✓	-	✓	✓	✓	✓	-	✓	✓	✓	✓	-	✓	-	-
08	✓	✓	✓	-	-	-	-	✓	✓	✓	✓	✓	✓	✓	✓	-	✓	-	✓	✓	-	✓	-	-	-
09	✓	✓	✓	-	-	-	-	✓	✓	-	✓	✓	✓	✓	-	✓	✓	-	✓	-	✓	✓	-	-	-
10	✓	-	✓	-	-	-	-	-	✓	✓	✓	-	✓	✓	✓	✓	✓	✓	✓	✓	✓	✓	✓	-	-
11	✓	✓	-	-	-	-	-	-	✓	-	✓	✓	-	✓	-	✓	✓	-	✓	-	✓	✓	-	-	-
12	✓	✓	✓	-	-	-	-	-	-	-	✓	✓	✓	-	-	✓	✓	✓	-	-	✓	✓	✓	-	-
13	✓	✓	✓	-	-	-	-	✓	✓	✓	✓	✓	✓	✓	✓	✓	✓	-	✓	✓	✓	✓	-	-	-
14	✓	✓	✓	-	-	-	-	✓	-	-	✓	✓	✓	-	-	✓	✓	-	-	-	✓	✓	-	-	-
15	✓	-	-	-	-	-	-	-	✓	✓	✓	-	-	✓	✓	-	-	-	✓	✓	-	-	-	-	-
16	✓	-	✓	-	-	-	-	✓	✓	✓	✓	-	✓	✓	✓	✓	-	✓	✓	✓	✓	-	✓	-	-
17	✓	✓	✓	-	-	-	-	✓	✓	-	✓	✓	✓	✓	-	✓	✓	✓	✓	-	✓	✓	✓	-	-
18	✓	✓	-	-	-	-	-	✓	✓	✓	✓	✓	✓	✓	✓	✓	✓	✓	✓	✓	-	✓	✓	-	-
19	✓	✓	-	-	-	-	-	✓	✓	-	✓	✓	✓	✓	-	✓	✓	✓	✓	-	✓	✓	✓	-	-
20	✓	-	-	-	-	-	-	-	-	✓	✓	-	-	-	✓	-	-	-	-	✓	-	-	-	-	-
21	✓	-	-	-	-	-	-	✓	-	-	✓	-	✓	-	-	✓	-	✓	-	-	✓	-	-	-	-
